# Gender Differences in 1-Year Clinical Characteristics and Outcomes after Stroke: Results from the China National Stroke Registry

**DOI:** 10.1371/journal.pone.0056459

**Published:** 2013-02-13

**Authors:** Zhan Wang, Jingjing Li, Chunxue Wang, Xiaomei Yao, Xingquan Zhao, Yilong Wang, Hao Li, Gaifen Liu, Anxin Wang, Yongjun Wang

**Affiliations:** 1 Department of Neurology, Beijing Tiantan Hospital, Capital Medical University, Beijing, China; 2 Department of Oncology, McMaster University, Ontario, Canada; FuWai Hospital, Chinese Academy of Medical Sciences, China

## Abstract

**Background:**

Previous reports have shown inconsistent results on clinical outcomes between women and men after stroke, and little is known about gender differences on outcomes in Chinese post-stroke patients. The aim of this study was to explore whether there were gender differences on clinical characteristics and outcomes in Chinese patients after ischemic stroke by using the data from the China National Stroke Registry (CNSR).

**Methods and Findings:**

Out of 12,415 consecutively recruited patients with acute ischemic stroke in the CNSR from 2007 to 2008, 11,560 (93.1%) patients were followed up for 12 months. Their clinical characteristics and outcomes on death, recurrence, and dependency were recorded. The multivariate logistic regression was performed to determine whether there were gender differences in these outcomes. Women were older than men at baseline (67.9 vs. 64.0 years, *P*<0.001). Women had a higher mortality, recurrence rate, and dependency rate at 3, 6, and 12 months than men, but after adjusting for age, history of diabetes, pre-stroke dependency, stroke severity, in-hospital complications, and other confounders, there were no statistically significant differences in gender on mortality and recurrence rate at 3, 6, and 12 months; and dependency rate at 3, and 6 months. However, the dependency rate at 12 months remained significantly higher in women (odds ratio, 1.24; 95% confidence interval, 1.06 to 1.45).

**Conclusions:**

There are many differences in clinical characteristics between women and men after ischemic stroke in China. Compared with men, women are more dependent at 12 months after stroke. This difference still exists after controlling the potential confounders.

## Introduction

Stroke is one of the leading causes of death and disability throughout the world [1]. The incidence and burden of stroke in China is increasing rapidly over time just like in other developing countries. It is now becoming the first leading cause of death in China [2]. Gender differences on prognostic outcomes in stroke patients have been explored in many countries. However, the results from these studies are inconsistent. Some studies showed that women had poorer prognoses [3], [4], [5], [6], [7], but some studies reported no gender differences on survival rate [8], [9], and others even demonstrated that women had better outcomes than men after stroke [10], [11], [12]. These differences among the previous studies may be relevant to the different patient selection criteria and follow-up periods (most studies had a less than one-year follow-up). Furthermore, most studies included patients with all types of stroke (i.e., ischemic stroke, intracerebral hemorrhage, and subarachnoid hemorrhage [SAH]), which would make it hard to interpret the results because gender differences already exist in the different types of stroke.

It has been realized that very few studies have investigated the gender differences on stroke in Chinese patients, especially for the prognostic outcomes [13]. The China National Stroke Registry (CNSR) was a population-based, large-scale prospective registry, which covered all 27 provinces and 4 municipalities in China including the Hong Kong region. The more details of the study design for the CNSR have been published elsewhere [14]. As a part of the CNSR, this study only focused on patients with ischemic stroke and with completed follow-up data for one year. The purpose of this study was to determine whether there were gender differences on clinical characteristics and 3-, 6- and 12-month outcomes in Chinese patients after ischemic stroke.

## Materials and Methods

### Subjects

**Figure 1:**
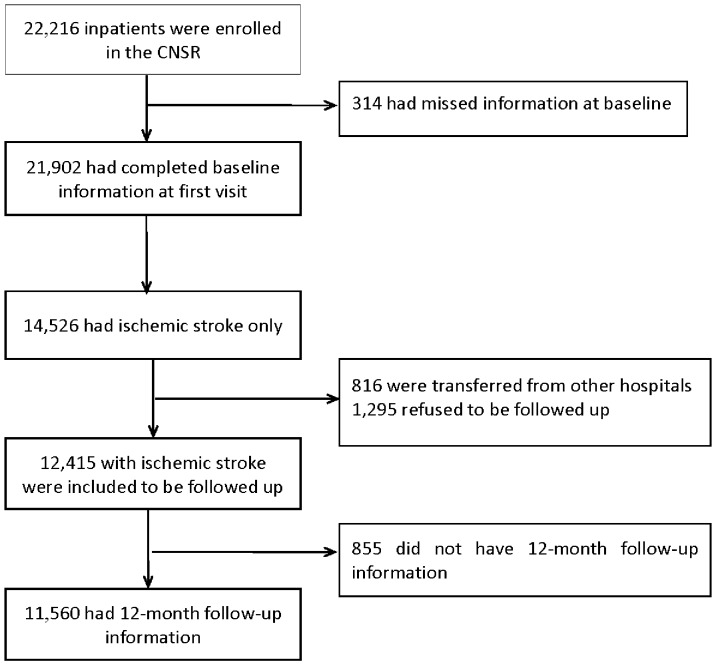
Flow diagram of participants.

The CNSR prospectively recruited consecutive stroke patients from 132 tertiary or secondary hospitals in China from September 2007 to August 2008. Patients were eligible to enroll if they were 18 years of age or older with stroke symptom onset within 14 days, and diagnosed as stroke or transient ischemic attack (TIA). Stroke was defined as acute ischemic stroke, intracerebral hemorrhage, and SAH, with brain computed tomography (CT) or magnetic resonance (MR) confirmation, which was consistent with the diagnostic criteria from World Health Organization [15]. Except for the patients who refused to participate in the survey, a total of 22,216 patients were enrolled in the CNSR and 14,526 of them had ischemic stroke. Among 14,526 patients with ischemic stroke, 816 patients who were transferred from other non-enrolled hospitals were excluded from our study because the treatments in other hospitals would affect the prognosis of stroke. Also, 1,295 patients who refused to be followed up were excluded from our study. Thus, 12,415 patients with ischemic stroke who had completed baseline information at the first visit were included in our study. After a one-year follow-up, the completed data from 11,560 patients (93.1%) were obtained (Figure 1). The study was approved by the ethics committees at all participating hospitals and the written informed consents were obtained from all participants or their designated relatives.

### Patient characteristics

In the current study, patient characteristics information at admission was collected, including age, sex, pre-stroke dependency, social-economic factors, and potential risk factors for stroke. The pre-stroke dependency was defined as the modified Rankin Scale (mRS) score of 3–5. The social-economic factors included marital status, patients' living arrangement before admission, education, and health insurance. The marital status included two types: married, and single/divorced/widowed/remarried. The patients' living arrangements were grouped into three categories: living with other family members, alone, and in a nursing home. The educational background was grouped into three types: elementary or below, middle school, and high school or above. The patients' health insurance was classified into four groups based on the national conditions in China [16], [17], [18]: (1) the Basic Health Insurance Scheme for the employed urban residents, which was jointly funded by the government, employers, and employees; (2) the New Cooperative Medical System provided by the Chinese government for rural residents is a communal system aiming at major medical treatment; (3) self-paid (no insurance), and (4) commercial or other insurances.

The potential risk factors included body mass index (BMI, calculated as weight in kilograms [kg] divided by height in meters squared [m^2^]); smoking status (smokers were defined as patients who were previous or present smokers); moderate or heavy alcohol (defined as more than 2 standard alcohol consumption per day [1 standard alcohol consumption equal to 120 ml wine, 360 ml beer, or 45 ml white spirit]). The potential risk factors also included the common risk factors of ischemic stroke, including the history of hypertension (defined as a self-reported history or anti-hypertensive drug use), diabetes mellitus (a self-reported history or current hypoglycemic medications use), dyslipidemia (a self-reported history or lipid-lowering drug use), coronary heart disease (a self-reported history or an angina history confirmed by a cardiologist in prior clinic visits), atrial fibrillation (a self-reported history with at least one confirmed electrocardiogram), and prior stroke (medical chart confirmed).

Additionally, the blood glucose, systolic and diastolic blood pressure (SBP and DBP), and the stroke severity (measured by the National Institute of Health Stroke Scale [NIHSS] [19] and Glasgow Coma Scale [GCS] [20]) at admission; the in-hospital complications; and thrombolysis treatment within three hours from the beginning of stroke symptom were also recorded.

### Outcomes

Death, recurrence, and dependency at 3, 6, and 12 months after stroke were assessed through telephone follow-ups. Recurrence was self-reported and defined as the aggravation of the original symptoms, new signs, or re-hospitalization with the diagnosis of ischemic stroke, intracerebral hemorrhage, or SAH. For patients who admitted hospitals again due to recurrent stroke, the information of diagnosis and classification of recurrent stroke were obtained from contacting the discharge department in the hospitals. Dependency was defined as the mRS score of 3–5. Mortality was defined as the cumulative death rate at 3, 6, and 12 months from the onset of stroke for any reason of death. The recurrence and dependency rates were defined as the cumulative rates at 3, 6, and 12 months after ischemic stroke, respectively. The standard scripts were used to collect the data of outcomes by the trained research personnel at Beijing Tiantan Hospital. The enrolled patients were the targeted respondents for the questionnaire. When it was impossible to talk with an enrolled patient or when the interviewer considered that the information provided by the patient was unreliable, his/her care-giver was contacted and interviewed. More details about the outcome assessment can be found in the protocol of the CNSR [14].

### Statistical analysis

The t test, Wilcoxon test, and chi-square test were used for univariate comparisons between genders, continuous variables in normal and abnormal distribution, and the categorical variables, respectively. The multivariate logistic regression was used to evaluate whether any of the patient characteristics (mentioned in the last section) might affect mortality, recurrence rate, and dependency rate at 12 months after onset of stroke. All data were analyzed using SAS version 9.2 statistical software and a two-sided significance level of α = 0.05 was assumed. The data analysts were blinded to the patients' gender information.

## Results

### Clinical characteristics between enrolled and excluded patients with ischemic stroke

Table 1 shows the baseline characteristics of the 11,560 enrolled patients and 2,966 excluded patients. The comparative results demonstrated that there was no significant difference in gender (61.6% versus 63.4%, *P* = 0.070), but the enrolled patients were two years older than the excluded patients on average (67.0 versus 65.0 years, *P*<0.001). The enrolled patients had a higher percentage of having a history of hypertension, dyslipidemia, prior stroke, atrial fibrillation, or coronary heart disease; a lower percentage of being married; a higher percentage of Basic Health Insurance Scheme; and a higher percentage of complications of new stroke, new atrial fibrillation, pneumonia, and stress ulcer than the excluded patients. There was no statistical difference for the severity of stroke between the enrolled and excluded patients.

**Table 1:** Baseline characteristics of enrolled and excluded patients with ischemic stroke.

Variables		Enrolled patients (n = 11,560)	Excluded patients* (n = 2,966)	*P* Value
**Gender**	men	7,118 (61.6%)	1,880 (63.4%)	0.070
	women	4,442 (38.4%)	1,086 (36.6%)	
**Mean age (range), years**		67.0 (57.0–75.0)	65.0 (54.0–74.0)	<0.001
**Pre-stroke dependency**		595 (5.1%)	148 (5.0%)	0.729
**Social-economic factors:**				
marital status	married	10,320 (89.7%)	2,677 (91.1%)	0.020
	single/divorced/widowed/remarried	1,185 (10.3%)	260 (8.9%)	
education	elementary or below	5,300 (45.9%)	1,246 (45.1%)	0.112
	middle school	2,976 (25.7%)	764 (27.6%)	
	high school or above	3,284 (28.4%)	754 (27.3%)	
health insurance	Basic Health Insurance Scheme	7,027 (60.8%)	1,496 (50.4%)	<0.001
	New Cooperative Medical System	1,953 (16.9%)	629 (21.2%)	
	self-paid	378 (3.3%)	129 (4.3%)	
	others (including commercial)	2,202 (19.1%)	712 (24.0%)	
**Risk factors of stroke:**				
BMI at admission	BMI<25 (kg/m^2^)	6,322 (60.6%)	1,505 (63.5%)	0.031
	25≤BMI<30 (kg/m^2^)	3,537 (34.0%)	741 (31.3%)	
	BMI≥30 (kg/m^2^)	559 (5.4%)	124 (5.2%)	
smoking		4,586 (39.7%)	1,187 (40.7%)	0.295
moderate or heavy alcohol		1,082 (9.4%)	278 (9.4%)	0.983
history of hypertension		7,401 (64.0%)	1,786 (60.2%)	<0.001
history of diabetes mellitus		2,503 (21.6%)	595 (20.1%)	0.059
history of dyslipidemia		1,312 (11.3%)	248 (8.4%)	<0.001
history of coronary heart disease		1,685 (14.6%)	331 (11.2%)	<0.001
history of atrial fibrillation		875 (7.6%)	169 (5.7%)	<0.001
history of prior stroke		3,967 (34.3%)	894 (30.1%)	<0.001
**Stroke severity:**				
NIHSS at admission	0–4	5,734 (49.6%)	1,477 (49.8%)	0.062
	5–14	4,384 (37.9%)	1,164 (39.2%)	
	≥15	1,442 (12.5%)	325 (11.0%)	
GCS at admission	= 15	7,459 (69.1%)	1,963 (71.3%)	0.070
	13–14	1,083 (10.0%)	273 (9.9%)	
	9–12	1,187 (11.0%)	284 (10.3%)	
	3–8	1,070 (9.9%)	234 (8.5%)	
**In-hospitalization complications:**				
new stroke		452 (3.9%)	50 (1.7%)	<0.001
seizure		131 (1.1%)	26 (1.0%)	0.312
urinary tract infection		457 (3.9%)	87 (2.9%)	0.009
pneumonia		1,373 (11.9%)	275 (9.3%)	<0.001
gastrointestinal hemorrhage		317 (2.7%)	42 (1.4%)	<0.001
decubitus ulcer		93 (0.8%)	20 (0.7%)	0.584
deep venous thrombosis		51 (0.4%)	12 (0.4%)	0.787
pulmonary embolism		39 (0.3%)	13 (0.4%)	0.412
myocardial infarct		75 (0.7%)	16 (0.5%)	0.501
atrial fibrillation		899 (7.8%)	175 (5.9%)	<0.001

BMI: body mass index, NIHSS: National Institute of Health Stroke Scale, GCS: Glasgow Coma Scale.

* The excluded patients with ischemic stroke included: 816 patients who were transferred from the other hospitals, 1295 patients who refused to participate in the follow-ups, and 855 patients who were lost to follow-up.

### Clinical characteristics between women and men

Among 11,560 ischemic stroke patients, 4442 (38.4%) were women. On average, women were 3.9 years older than men (mean 67.9 versus 64.0 years, *P*<0.001) in Table 2. Before stroke, women lived alone more (4.7% versus 2.8%, *P*<0.001), were single or divorced more (16.4% versus 6.5%, *P*<0.001), had a higher proportion of self-paid insurance (23.4% versus 16.3%, *P*<0.001) and a lower educational level (62.2% versus 35.6%, *P*<0.001) than men. Compared with men, women also had a higher proportion of pre-stroke dependency (6.0% versus 4.6%, *P*<0.001).

**Table 2:** Baseline characteristics of different gender patients with ischemic stroke.

Variables		Women (n = 4,442)	Men (n = 7,118)	*P* Value
**Age(X±SD)(years)**		67.9±11.9	64.0±12.3	<0.001
**Pre-stroke dependency**		267 (6.0%)	328 (4.6%)	<0.001
**Social-economic factors:**				
marital status	married	3,691(83.6%)	6,629 (93.5%)	<0.001
	single/divorced/widowed/remarried	725 (16.4%)	460 (6.5%)	
living arrangement	with other family members	4,160 (94.7%)	6,850 (97.0%)	<0.001
	alone	208 (4.7%)	197 (2.8%)	
	nursing home	26 (0.6%)	18 (0.2%)	
education	elementary or below	2,763 (62.2%)	2,537 (35.6%)	<0.001
	middle school	878 (19.8%)	2,098 (29.5%)	
	high school or above	801 (18.0%)	2,483 (34.9%)	
health insurance	Basic Health Insurance Scheme	2,339(52.7%)	4,688 (65.9%)	<0.001
	New Cooperative Medical System	893 (20.1%)	1,060 (14.9%)	
	self-paid	1,040 (23.4%)	1,162 (16.3%)	
	others (including commercial)	170 (3.8%)	208 (2.9%)	
**Risk factors of stroke:**				
BMI at admission	BMI<25 (kg/m^2^)	2,358 (59.5%)	3,964 (61.4%)	<0.001
	25≤BMI<30 (kg/m^2^)	1,342 (33.8%)	2,195 (34.0%)	
	BMI≥30 (kg/m^2^)	266 (6.7%)	293 (4.6%)	
smoking		268 (6.0%)	4,318 (60.7%)	<0.001
moderate or heavy alcohol		22 (0.5%)	1,060 (14.9%)	<0.001
history of hypertension		3,026 (68.1%)	4,375 (61.5%)	<0.001
history of diabetes mellitus		1,102 (24.8%)	1,401 (19.7%)	<0.001
history of dyslipidemia		490 (11.0%)	822 (11.5%)	0.394
history of coronary heart disease		769 (17.3%)	916 (12.9%)	<0.001
history of atrial fibrillation		494 (11.1%)	381 (5.3%)	<0.001
history of prior stroke		1,509 (34.0%)	2,458 (34.5%)	0.537
**Blood glucose at admission(X±SD)(mmol/L)**		6.94±2.43	6.67±2.30	<0.001
**SBP at admission(X±SD)(mmHg)**		151.7±24.9	150.1±23.7	0.057
**DBP at admission(X±SD)(mmHg)**		86.9±13.9	88.5±14.0	<0.001
**Stroke severity:**				
NIHSS at admission	0–4	2,011 (45.3%)	3,723 (52.3%)	<0.001
	5–14	1,736 (39.1%)	2,648 (37.2%)	
	≥15	695 (15.6%)	747 (10.5%)	
GCS at admission	= 15	2,757 (66.3%)	4,702 (70.8%)	<0.001
	13–14	415 (10.0%)	668 (10.1%)	
	9–12	504 (12.1%)	683 (10.3%)	
	3–8	485 (11.7%)	585 (8.8%)	
**Onset to arrival time(0∼3 hours)**		840 (22.3%)	1,279 (21.2%)	0.188
**Thrombolysis given**		156 (4.2%)	269 (4.5%)	0.337
**In-hospitalization complications:**				
new stroke		190 (4.3%)	262 (3.7%)	0.108
seizure		63 (1.4%)	68 (1.0%)	0.022
urinary tract infection		285 (6.4%)	172 (2.4%)	<0.001
pneumonia		578 (13.0%)	795 (11.2%)	0.003
gastrointestinal hemorrhage		115 (2.6%)	202 (2.8%)	0.425
decubitus ulcer		51 (1.2%)	42 (0.6%)	0.001
deep venous thrombosis		26 (0.6%)	25 (0.4%)	0.065
pulmonary embolism		19 (0.4%)	20 (0.3%)	0.186
myocardial infarct		33 (0.7%)	42 (0.6%)	0.319
atrial fibrillation		523 (11.8%)	376 (5.3%)	<0.001

X: mean, SD: standard deviation, BMI: body mass index, SBP: systolic blood pressure, DBP: diastolic blood pressure, NIHSS: National Institute of Health Stroke Scale, GCS: Glasgow Coma Scale.

Women were significantly more likely to have histories of hypertension, diabetes, coronary heart disease, and atrial fibrillation than men, and had a significantly higher frequency of overweight (6.7% versus 4.6% for BMI≥30 kg/m^2^), but were significantly less likely to smoke and/or to have moderate or heavy alcohol. The level of blood glucose at admission was higher in women than in men as well as DBP, but SBP was not significantly different between them.

Table 2 also shows that women had severer stroke and were more likely to be comatose than men. There were no gender differences in the proportion of patients with thrombolysis treatment (4.2% versus 4.5% for women versus men, *P* = 0.337), and in the proportion of patients presenting to hospital within three hours of symptom recognition (22.3% versus 21.2%, *P* = 0.188). During the hospitalization, women were significantly more likely to experience seizure, urinary tract infection, pneumonia, decubitus ulcer, and new atrial fibrillation.

### Outcomes at 3, 6, and 12 months after stroke onset

Without controlling potential confounders, the outcomes of mortality, recurrence rate, and dependency rate at 3, 6, and 12 months after stroke are shown in Table 3. The multivariate logistic regression analysis showed that age, history of diabetes, history of stroke, pre-stroke dependence, stroke severity, blood glucose at admission, and the complications of pneumonia and new atrial fibrillation had significant association with mortality, recurrence rate, and dependency rate at 12 months (Table 4). The mortality was significantly higher in post-stroke women than those in men at 3, 6, and 12 months, respectively in Table 3 (11.3% versus 7.6%, 14.4% versus 9.7%, 17.8% versus 12.3%; all *P*<0.001), but after adjusting for the above confounding factors, there were no gender differences on mortality at 3, 6, and 12 months in Table 5 (adjusted odds ratio [OR] 1.06, 1.10, 1.09, respectively; all *P*>0.05).

**Table 3:** Clinical outcomes at 3, 6, and 12 months after ischemic stroke.

Outcomes*	3 months			6 months			12 months		
	Women	Men	*P* Value	Women	Men	*P* Value	Women	Men	*P* Value
**Mortality (n/N, %)**	499/4,437 (11.3%)	538/7,113 (7.6%)	<0.001	639/4,424 (14.4%)	685/7,078 (9.7%)	<0.001	789/4,442 (17.8%)	875/7,118 (12.3%)	<0.001
**Recurrence rate (n/N, %)**	641/4,442 (14.4%)	853/7,118 (12.0%)	<0.001	804/4,437 (18.1%)	1,044/7,103 (14.7%)	<0.001	883/4,442 (19.9%)	1,167/7,118 (16.4%)	<0.001
**Dependency rate (n/N, %)**	1,345/3,943 (34.1%)	1,607/6,580 (24.4%)	<0.001	1,169/3,803 (30.7%)	1,440/6,433 (22.4%)	<0.001	1,049/3,653 (28.7%)	1,192/6,243 (19.1%)	<0.001

* Mortality was defined as the cumulative death rate at 3, 6, and 12 months from the onset of stroke for any reason of death. Recurrence was defined as the aggravated primary neurological deficit, new signs, or rehospitalization with the diagnosis of ischemic stroke, intracerebral hemorrhage, or subarachnoid hemorrhage. Dependency was defined as the modified Rankin Scale scores of 3–5. The recurrence and dependency rates were defined as the cumulative rates at 3, 6, and 12 months after ischemic stroke, respectively.

**Table 4:** Multivariate logistic regression analysis for confounders and outcomes at 12 months after stroke onset.

Variables	Mortality OR(95%CI)	Recurrence rate OR(95%CI)	Dependency rate OR(95%CI)
**Age**	1.05(1.04–1.05)	1.02(1.01–1.02)	1.04(1.04–1.05)
**Pre-stroke dependency**	1.49(1.18–1.88)	1.65(1.36–2.01)	2.13(1.69–2.67)
**History of smoking**	0.87(0.72–1.04)*	0.93(0.81–1.07)*	0.74(0.64–0.86)
**History of diabetes**	1.30(1.09–1.54)	1.26(1.08–1.47)	1.42(1.19–1.69)
**History of dyslipidemia**	0.55(0.41–0.75)	0.92(0.74–1.13)*	0.89(0.72–1.11)*
**History of coronary heart disease**	0.96(0.77–1.20)*	1.20(1.01–1.43)	0.98(0.81–1.20)*
**History of atrial fibrillation**	1.41(1.07–1.89)	1.06(0.81–1.37)*	0.98(0.71–1.37)*
**History of prior stroke**	1.36(1.14–1.62)	1.43(1.24–1.64)	1.31(1.13–1.53)
**Glucose at admission**	1.08(1.05–1.11)	1.02(0.99–1.04)*	1.04(1.01–1.08)
**NIHSS at admission**			
≥15 versus 0–4	5.26(4.02–6.88)	1.79(1.41–2.28)	11.38(8.48–15.27)
5–14 versus 0–4	1.55(1.26–1.90)	1.18(1.01–1.37)	2.97(2.54–3.46)
**GCS at admission**			
3–8 versus 15	2.54(1.96–3.29)	1.37(1.09–1.73)	1.68(1.29–2.17)
9–12 versus 15	1.83(1.42–2.36)	1.30(1.04–1.63)	1.41(1.10–1.79)
13–14 versus 15	1.57(1.21–2.05)	1.07(0.85–1.34) *	1.32(1.06–1.64)
**Pneumonia complication**	2.75(2.26–3.35)	1.71(1.43–2.05)	1.86(1.48–2.33)
**New atrial fibrillation complication**	1.45(1.09–1.93)	1.61(1.29–1.99)	1.36(1.04–1.79)

OR: odds ratio; CI: confidence interval; NIHSS: National Institute of Health Stroke Scale; GCS: Glasgow Coma Scale.

* Indicated not significantly different.

**Table 5:** Unadjusted and adjusted OR of outcomes at 3, 6, and 12 months for women to men.

Outcomes		Unadjusted OR (95%CI)	Adjusted OR (95%CI) *
**Mortality**	at 3 months	1.55(1.36–1.76)†	1.06(0.91–1.24)
	at 6 months	1.58(1.41–1.77)†	1.10(0.95–1.27)
	at 12 months	1.54(1.39–1.71)†	1.09(0.95–1.24)
**Recurrence rate**	at 3 months	1.24(1.11–1.38)†	1.03(0.91–1.16)
	at 6 months	1.28(1.16–1.42)†	1.06(0.95–1.19)
	at 12 months	1.26(1.15–1.39)†	1.05(0.94–1.17)
**Dependency rate**	at 3 months	1.60(1.47–1.75)†	1.15(0.99–1.34)
	at 6 months	1.54(1.41–1.68)†	1.13(0.97–1.31)
	at 12 months	1.71(1.55–1.88)†	1.24(1.06–1.45)†

OR: odds ratio.CI: confidence interval.

* The adjusted risk factors are age, history of diabetes, history of stroke, pre-stroke dependency, stroke severity and blood glucose at admission, and the complications of pneumonia and new atrial fibrillation.

† P<0.05.

A similar pattern was observed for recurrence rate. Before the adjustment of the above confounding factors, women's recurrence rates were significantly higher than men's at 3, 6, and 12 months after stroke onset (14.4% versus 12.0%, 18.1% versus 14.7%, 19.9% versus 16.4%, respectively, all *P*<0.001 in Table 3). There were no more significant differences between them after the adjustment (adjusted OR 1.03, 1.06, 1.05, respectively; all *P*>0.05 in Table 5).

The unadjusted dependency rates were significantly higher in women than those in men at 3, 6, 12 months after stroke (34.1% versus 24.4%, 30.7% versus 22.4%, 28.7% versus 19.1%, respectively; all *P*<0.001 in Table 3) and no more significant differences were observed after adjusting for the confounders (adjusted OR 1.15, 1.13, respectively; all *P*>0.05 in Table 5). However, women were more likely to be dependent than men at 12 months even after adjusting for the confounders (adjusted OR, 1.24; 95% confidence interval [CI], 1.06 to 1.45, *P*<0.05 in Table 5).

## Discussion

The CNSR was a prospective stroke registry that had extensive coverage in China and involved the maximal stroke patients in China so far. In the current study, patients with ischemic stroke from the CNSR were selected, and the gender differences on the outcomes of mortality, recurrence rate and dependency rate were analyzed. Several previous studies [13], [21], [22], [23], [24] reported inconsistent results of the gender differences on prognoses in patients with ischemic stroke. Therefore, it prompted us to focus on the gender differences on prognoses in patients with ischemic stroke only in this study. Moreover, many previous studies [3], [4], [6], [8], [9], [11], [25], [26] aiming at gender differences in patients with all types of stroke demonstrated that there were gender differences in different stroke types. For example, the results in the Sheikh et al study [10] showed that after adjusting for age, there was no sex difference in 1-year mortality between men and women with hemorrhagic stroke (including subarachnoid hemorrhage and intracerebral hemorrhage), but men had a higher mortality than women with ischemic stroke after 1-year follow-up. Thus, we put great effort to study the gender differences on prognoses in the subgroup of ischemic stroke patients.

Among the enrolled patients with ischemic stroke, we found that women were more likely to have risk factors of stroke that is different from some published literatures in Chinese populations. For example, the 2007–2008 China National Diabetes and Metabolic Disorders Study [27] reported that the prevalences of hypertension and diabetes were higher in men than those in women who were in the primary prevention (patients had risk factors but did not develop stroke). Differing from this kind of study, our study mainly focused on patients with ischemic stroke. There were also a few studies that demonstrated all of the risk factors of stroke were significantly higher in men than in women with stroke. For instance, the Zhang et al study [28] found that young men with stroke (aged 18–45 years) were more likely to have hypertension, diabetes mellitus, and hyperlipidemia than women. However, the enrolled patients in our study were older (67.9 years for women and 64.0 years for men) than those in the Zhang et al study. The different results in risk factors could be explained by the differences in age and target populations. Similar to our findings, the Kong et al [13] and the Huang et al [29] studies showed that women were more likely to have diabetes mellitus and hypertension than men with ischemic stroke.

In our results, there were gender differences for mortality, recurrence rate and dependency rate at 3, 6, and 12 months after stroke onset. After adjusting for the confounders, the gender differences disappeared for mortality and recurrence rate. There were no gender differences for dependency rates at 3 and 6 months. However, at 12 months after stroke onset, women were still more disabled than men.

Many reports from different countries have assessed sex differences on mortality in stroke patients and their results remain surprisingly variable. Some reports showed no difference for mortality after stroke [8], [9], [30], [31], [32], some showed that women had a higher mortality than men [4], [6], and others showed a lower mortality in women [10], [11], [12], [21], [25]. Before adjusting for the confounders, our study showed a higher mortality in women than that in men. However, the difference was not significant after controlling potential confounding factors such as age, history of diabetes, and so on. This suggests that a higher mortality in post-stroke women is largely attributed to their older age, more pre-stroke dependency, severer neurological defect, more cardiovascular risk factors, and a higher pneumonia complication, but not their gender. The Korean Stroke Registry [21], which included 18,634 ischemic stroke or TIA patients admitted within 7 days after symptom onset, showed the similar results as our study at 14-day and 25-month follow-up before the adjustment. But after adjusting for baseline characteristics of age, histories of hypertension and diabetes mellitus, neurological severity at admission and other factors, women had a greater survival rate than men during both the short- and long-term periods after stroke. The same result was seen in the Demark national registry [25] before the adjustment, which showed a higher crude mortality in women at 30 and 90 days after stroke. But a lower mortality appeared in women after adjusting for the clinical characteristics and other confounders. These different results among the studies probably reflect differences in race/ethnicity and/or follow-up time. Additionally, whether controlling the potential confounders and/or what kind of the confounding factors were controlled also resulted in different results in mortality after stroke.

Despite the fact that a few studies have been done to find the gender differences in functional outcomes after stroke, almost all the published studies from Europe, North America, and Asia have shown poorer functional outcomes in women than those in men [8], [9], [13], [21], [22], [23], [24], [26], [32], [33], [34]. Di Carlo A [32] and his colleagues found that women were more disabled and more dependent than men at 3 months after stroke. Their data were from a large registry of multicentres in many countries in Europe and all types of stroke were included. The Rankin Scale was used to evaluate the outcome of handicap at 3-month follow-up and it was significantly higher in women than that in men (2.6±1.5 versus 2.2±1.5, *P*<0.001; Mann-Whitney test). In a Paul Coverdell National Acute Stroke Registry (PCNASR, a statewide stroke registry in America), women were less likely to achieve activities of daily living independence (defined as the Barthel Index≥95) even after adjustment for potential confounders (adjusted OR: 0.37, 95% CI: 0.19 to 0.87), and the researchers also reported the lower least-squares means Stroke-Specific Quality of Life scores in women than that in men [33]. The results from the Registry of the Canadian Stroke Network revealed that women had a slightly worse functional status at 6 months after stroke than men, when they were measured by the Stroke Impact Scale-16 [9]. Our findings are consistent with these reports of gender differences in functional outcomes although we used different measurements to estimate the outcomes and followed patients longer (one year). It has not yet been fully explained why women have poorer functional outcomes. Reeves et al [35] considered the fact that women were more likely to be widowed and had less social support than men might be the reasons for women's poor functional outcomes. However, in our study, the social factors, such as living arrangement, marriage, education, and health insurance, had no significant impact on mortality, recurrence rate, and dependency rate after a multivariate regression analysis. These social factors may prevent women from having good functional outcomes, but their influence is too small to be emphasized when compared with age, stroke severity, history of diabetes mellitus, and pre-stroke function.

Recurrence at 3, 6, and 12 months were similar to death outcome in this study. Nevertheless, in Japan, Fukuda et al [36] found no gender differences on stroke recurrence rate at 1 and 5 years even before controlling potential confounders.

Interestingly, the history of diabetes not hypertension had significant influence on mortality, recurrence rate, and dependency rate in our study. Data from Qian et al [37] and Kong et al [13] confirmed that the post-stroke patients with diabetes mellitus would have poorer outcomes. Although some of previous studies showed that women were less likely to have treatment of thrombolysis than men [7], [21], [38], [39], [40], our results showed no gender difference for this treatment, which is consistent with other previous reports [6], [9], [41]. The similar time to arrive at the hospitals between women and men (in three hours after symptom onset) might be the reason for this point in our study.

### Strengths and limitations

This study is a part of the largest population-based prospective stroke registry in China. The completed data with one-year follow-up have enabled us to investigate the relationship between gender differences and prognoses in post-stroke patients in China, which has not been well-explored previously. However, there are some limitations in this study. Firstly, the enrolled patients were restricted to those from the teaching hospitals or higher level non-teaching hospitals in urban areas of China, not recruiting community-level clinic centers, particularly not covering rural hospitals, where there are high stroke incidences and poor managements. Secondly, regarding risk factors for stroke in women, in addition to common classic risk factors, there are some specific risk factors that may be related to the prognoses of stroke, including pregnancy, delivery, childbearing, puerperium, oral contraceptives, migraine with aura, and hormone replacement therapy after menopause. These specific factors among the specific age groups have remained unclear in the study, which limits the underlying causes of findings in the specific age subgroups and needs further investigation. Thirdly, many patients with ischemic stroke (2,966 from 14,526) were excluded that might bias the results. Although there was no significant difference in gender between the enrolled and excluded patients, the excluded patients were younger and less likely to have the risk factors of ischemic stroke. Therefore, the outcomes of the excluded patients might be better than those of the enrolled patients. Fourthly, we did not analyze other types of stroke although their data were collected in the CNSR. In the future, studies are needed to explore the gender differences on prognoses in the patients with other types of stroke, such as hemorrhagic stroke. Fifthly, we followed up patients by telephone not by face to face interview because too many patients were enrolled in this large registry. Thus, the detailed data of stroke recurrence could not be collected in this study and the accuracy of these self-reported events needs to be evaluated. Finally, the Cox proportional hazard regression model may be properer than the logistic regression model to analyze data in our study. However, the precise follow-up data about death or recurrence time of patients were not collected during the telephone follow-ups. Although we used the logistic regression model to analyze data, which could not identify the correlation between the prognostic factors and time, it could still determine the impact from the risk factors on patients' prognoses after ischemic stroke during the follow-up time.

## Conclusions

Our present study indicates that women have many differences in clinical characteristics after ischemic stroke when compared with men in China. The crude mortality, recurrence rate, and dependency rate at 3, 6, and 12 months are higher in women than those in men after ischemic stroke, but the adjustments for the confounders eliminate those differences except for that the 12-month dependency rate is still higher in women. Future research should focus on exploring the reasons for this difference in dependency outcome.
