# Correction: Gender Differences in 1-Year Clinical Characteristics and Outcomes after Stroke: Results from the China National Stroke Registry

**DOI:** 10.1371/annotation/3ff065bf-8100-4ddd-be5b-88f81ffcfe04

**Published:** 2013-03-19

**Authors:** Zhan Wang, Jingjing Li, Chunxue Wang, Xiaomei Yao, Xingquan Zhao, Yilong Wang, Hao Li, Gaifen Liu, Anxin Wang, Yongjun Wang

In the Author Byline, the corresponding author was noted incorrectly. 

The correct corresponding author is Chunxue Wang, with the email address snow_sen@yahoo.com

